# Pangenome Evolution Reconciles Robustness and Instability of Rhizobial Symbiosis

**DOI:** 10.1128/mbio.00074-22

**Published:** 2022-04-13

**Authors:** Alexandra J. Weisberg, Arafat Rahman, Dakota Backus, Parinita Tyavanagimatt, Jeff H. Chang, Joel L. Sachs

**Affiliations:** a Department of Botany and Plant Pathology, Oregon State University, Corvallis, Oregon, USA; b Department of Evolution, Ecology and Organismal Biology, University of California Riverside, Riverside, California, USA; c Department of Microbiology and Plant Pathology, University of California Riverside, Riverside, California, USA; d Institute for Integrative Genome Biology, University of California Riverside, Riverside, California, USA; University of Pittsburgh

**Keywords:** evolution, symbiosis, robustness, nitrogen fixation, mobile genetic elements, rhizobia

## Abstract

Root nodulating rhizobia are nearly ubiquitous in soils and provide the critical service of nitrogen fixation to thousands of legume species, including staple crops. However, the magnitude of fixed nitrogen provided to hosts varies markedly among rhizobia strains, despite host legumes having mechanisms to selectively reward beneficial strains and to punish ones that do not fix sufficient nitrogen. Variation in the services of microbial mutualists is considered paradoxical given host mechanisms to select beneficial genotypes. Moreover, the recurrent evolution of non-fixing symbiont genotypes is predicted to destabilize symbiosis, but breakdown has rarely been observed. Here, we deconstructed hundreds of genome sequences from genotypically and phenotypically diverse *Bradyrhizobium* strains and revealed mechanisms that generate variation in symbiotic nitrogen fixation. We show that this trait is conferred by a modular system consisting of many extremely large integrative conjugative elements and few conjugative plasmids. Their transmissibility and propensity to reshuffle genes generate new combinations that lead to uncooperative genotypes and make individual partnerships unstable. We also demonstrate that these same properties extend beneficial associations to diverse host species and transfer symbiotic capacity among diverse strains. Hence, symbiotic nitrogen fixation is underpinned by modularity, which engenders flexibility, a feature that reconciles evolutionary robustness and instability. These results provide new insights into mechanisms driving the evolution of mobile genetic elements. Moreover, they yield a new predictive model on the evolution of rhizobial symbioses, one that informs on the health of organisms and ecosystems that are hosts to symbionts and that helps resolve the long-standing paradox.

## INTRODUCTION

A predictive understanding of symbiosis evolution is critical to inform on the health of hosts and ecosystems in which microbial symbionts reside. A central feature of symbiosis is the variation in the magnitude of services that symbionts provide (1). At one extreme are uncooperative strains, those that abandon intimate association with hosts or that are ineffective in providing benefits (2). Their recurrent origins are predicted to destabilize symbiosis, but breakdown has rarely been observed, presenting a paradox of instability and robustness (2, 3). Discovering mechanisms that generate and maintain variation in microbial symbionts is foundational for building a unifying framework for symbiosis evolution and resolving the paradox (1).

Genetic variation occurs through mutation, recombination, and gene flow, which together underlie the concept of the pangenome, a nonredundant set of genes in organisms related through ancestry and divisible into core and accessory genomes (4). The core represents genes predicted to encode functions common and essential among related strains. The accessory genome consists of genes polymorphic in presence/absence and confers upon related individuals the ability to adopt diverse lifestyles. In bacteria, mobile genetic elements (MGEs) are important molecules of accessory genomes. MGEs tend to be arranged into functional units, an organization that promotes modularity, a property that preserves functionality while allowing components to separate and recombine, thereby conferring flexibility and robustness to adapt to different conditions (5). Thus, MGEs are important to bacterial evolution because their exchangeability increases opportunities to recombine, reassort accessory cargo genes, and diversify. Resolving relationships of MGEs is essential for understanding the evolution of traits they encode.

Integrative conjugative elements (ICEs) and plasmids are two major classes of MGEs and both carry cargo genes that encode traits, such as virulence and antibiotic resistance, associated with transitions in the evolution of bacteria (6, 7). ICEs typically recombine into chromosomes and replicate passively, while plasmids typically replicate independently from the chromosome. ICEs encode integrases that can mediate site-specific recombination between homologous attachment (*att*) sequences located on the ICE and chromosome, which are often in a conserved gene, such as a tRNA gene. ICEs can also excise, circularize, mobilize one strand through a type IV secretion system (T4SS), and recombine into the genome of recipient strains and back into that of the donor strain. The identification of ICEs, and their distinction from nonmobile genomic islands, is confounded by challenges in determining *att* sites as well as by compositional variation and presence of many repeat sequences that fragment these elements (8). Consequently, fundamental aspects of the mechanisms that generate diversity and the extent of variation within related ICEs are poorly understood.

MGEs are crucial for the ability of many taxa of rhizobia to carry out symbiotic nitrogen fixation (SNF), a service essential to ecosystems (9). Beneficial rhizobia are defined by two sets of functions, the capacity to nodulate hosts and the ability to fix nitrogen, that are often encoded on MGEs. Symbiosis ICEs and plasmids have clusters of *nod*, *nol*, and *noe* genes (collectively *nod* genes here) for the synthesis of Nod factors, signaling molecules that initiate interactions and influence host specificity, as well as clusters of *nif/fix* genes for the catalysis of nitrogen fixation (10). Common NodABC proteins synthesize the core signaling structure while others, encoded by genes polymorphic in presence/absence, decorate the core with diverse substitutions (9). Type III secretion system (T3SS)- and effector-encoding genes are frequently linked to symbiosis genes (11). Effector genes influence host specificity because of their dichotomous abilities in dampening and inciting plant immune responses (12).

Symbiosis ICEs (symICEs) were first characterized in *Mesorhizobium* (10, 13, 14). *Mesorhizobium* symICEs adopt monopartite or polypartite structures, with the latter in *Mesorhizobium* consisting of three elements, each encoding their own integrase, that interact to circularize and mediate genomic rearrangements during integration or excision from the chromosome (13). In agronomic landscapes – where symICEs have been extensively studied – transfer of entire symICEs promotes diversification of *Mesorhizobium* strains that nodulate crop hosts, but with mixed results for effective symbiosis. For instance, in settings where legume crops and compatible rhizobia were introduced by growers, transfer of an entire symICE from highly effective inoculant strains to native rhizobia occurred and generated a diversity of novel nodulating strains; however, many of them were ineffective in fixing nitrogen and the bases for loss of SNF on target crops remain unknown (15–17). Moreover, processes that drive symICE variation, e.g., monopartite and polypartite and diverse integration sites, as well as the selective advantages for such variation in nitrogen fixing bacteria are also unknown (18, 19).

In contrast to other rhizobia, most members of *Bradyrhizobium* are traditionally thought to have genes necessary for SNF clustered in a genomic island referred to as a symbiosis island (SI) (20). *Bradyrhizobium* is cosmopolitan and its members can fix nitrogen in facultative associations with diverse members of the legume family, Fabaceae. (21). Host species include at least 24 of the 33 legume tribes that can form nodules, spread across the three legume subfamilies, Caesalpinioideae, Mimosoideae, and Papilionoideae. *Bradyrhizobium* populations have been extensively characterized in native host communities, and shown to exhibit broad variation in symbiotic capacity, providing natural tests to investigate genomic drivers of this variation (22). We compiled and analyzed a data set of genome sequences of genetically and phenotypically diverse strains of *Bradyrhizobium* (Data set S1; Extended Data sets S1–S3 available at https://github.com/osuchanglab/BradyrhizobiumPangenomeManuscript/tree/main/Extended_Supplementary_Materials). Critical to resolving the drivers of symbiosis variation in natural settings, we sequenced genomes of 85 strains (here metapopulation strains) isolated from across an 800 km transect of wild *Acmispon strigosus* populations in California and phenotyped the strains as beneficial (Nod^+^/Fix^+^), ineffective (Nod^+^/Fix-), or non-nodulating (Nod-/Fix-) on this host species (22, 23). We additionally included 167 publicly available genome sequences of strains beneficial to plants of many legume tribes as well as strains considered non-nodulating, photosynthetic, or not isolated from plants. Here, findings suggested that the SI of *Bradyrhizobium* represents a diverse set of symICEs. We additionally demonstrate that recombination among symICEs and with nonsymbiosis ICEs as well as nonsymbiosis plasmids generates tremendous structural and functional diversity. Modularity of genes that contribute to SNF and their presence on mobile genetic elements are key to generating variation and conferring robustness to this ecologically important trait.

10.1128/mbio.00074-22.8 DATA SET S1 Strain Metadata. Download Data Set S1, XLSX file, 0.03 MB. Copyright © 2022 Weisberg et al. 2022 Weisberg et al. https://creativecommons.org/licenses/by/4.0/ This content is distributed under the terms of the Creative Commons Attribution 4.0 International license.

## RESULTS

### Symbiosis genes of *Bradyrhizobium* cluster within a strikingly diverse set of mobile genetic elements.

**FIG 1:**
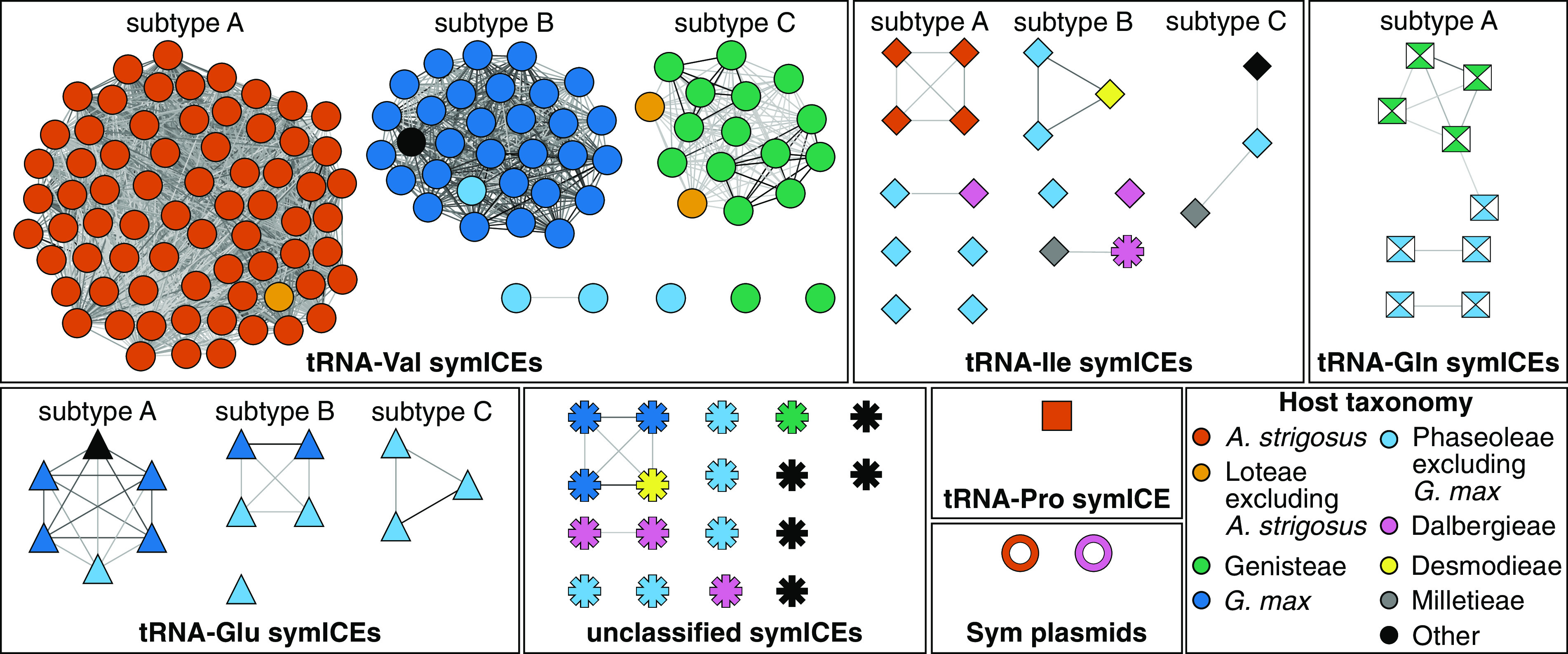
Symbiosis MGEs of *Bradyrhizobium* are diverse. (A) Weighted undirected network of symbiosis MGEs. Graphs are arranged according to type or class (shape) of symbiosis MGE. Graphs with more than two nodes were further classified into subtypes. Nodes represent individual symbiosis MGEs. Colors indicate taxonomic classification of associated host. Darker edges indicate greater Jaccard similarity of *k-*mer signatures.

We first identified clusters of symbiosis genes in *Bradyrhizobium* genome sequences and searched for hallmark features indicative of being within mobile genetic elements. A total of 179 strains have symbiosis genes clustered within regions with features of ICEs, here called symbiosis ICEs (symICEs), and two have them clustered within megaplasmids (Fig. 1). Both classes of genetic elements are associated with a T4SS-encoding locus that mediates interbacterial conjugation. In addition, the symICEs have integrase-associated genes, while the symbiosis megaplasmids (Sym plasmids) have a *repABC* origin of replication. The symICEs are also associated with tRNA genes and represent five types based on these associations and multiple subtypes based on differences in sequence signatures and gene composition (Fig. S1, S2A, S3; Data set S1). Analysis of replication genes suggest that the two Sym plasmids have independent origins (Fig. 1; Ext. Fig. S1 available at https://github.com/osuchanglab/BradyrhizobiumPangenomeManuscript/tree/main/Extended_Supplementary_Materials) (24). Twenty additional strains have *nif/fix* islands that lack features of ICEs or plasmids and two of these strains also have *nod* genes located in different regions originally classified as islands (25, 26). The remaining strains lack symbiosis genes and are incapable of SNF (Data set S1) (23).

10.1128/mbio.00074-22.1 FIG S1 Integrase genes of symICEs form multiple families. ML phylogenies of integrase gene families (A) *intV* (*att* sites are in tRNA-Val; *int* genes are in B elements), (B) *intY* (*att* sites are in *ybgC*; *int* genes are in tRNA-Val symICEs) and some homologs of *intI*, (C) *intH*, (D) *intGln* and a different lineage of *intI*, (E) *intGlu*, and (F) *intP*. (B) The phylogeny of *int* genes from tRNA-Val and tRNA-Ile symICEs suggests that *intY* and *intI* are distantly related and derived from a common ancestor. Black arrows highlight strains with representative integrase genes. (B) The island with *nod* and T3SS-encoding genes of strain ORS285 is associated with an integrase gene. (A-C) The tRNA-Val symICE variant of strain number 186 is a tripartite molecule and has three *int* genes. Clades are labeled according to the tRNA gene adjacent to the corresponding integrase gene. Those without labels are not located next to an identifiable tRNA gene. Trees are midpoint rooted. Branches colored black exceed 95% UFBoot and 80% SH-aLRT support. Scale bars indicate average number of substitutions per site. Tip points of the MLSA tree are colored according to the taxonomic classification of plant hosts and shaped according to the genomic location of the gene. Download FIG S1, PDF file, 0.1 MB. Copyright © 2022 Weisberg et al. 2022 Weisberg et al. https://creativecommons.org/licenses/by/4.0/ This content is distributed under the terms of the Creative Commons Attribution 4.0 International license.

10.1128/mbio.00074-22.2 FIG S2 Variations in presence/absence and number of T4SS- and T3SS-encoding loci. (A) ML phylogeny of translated sequences of *trbE,* a gene essential for type IV secretion system (T4SS)-mediated conjugation. TrbE is a member of the VirB4 family, a group of inner membrane associated ATPases. Most symbiosis MGEs isolated from *A. strigosus* have one copy of *trbE*; two have two copies. Conversely, the majority in strains isolated from *G. max* lack *trbE,* though it is possible that these symICEs can be mobilized in *trans*. Instances in which *trbE* gene are confidently associated with a plasmid (black arrows) are marked and suggests the T4SS of the Sym plasmid of strain number 187 was acquired from a symICE. The tree is midpoint rooted. (B) ML phylogeny of *sctU,* which encodes an inner membrane protein of the T3SS. Ineffective strain number 200 has a novel T3SS locus located in the chromosome and unique relative to the canonical and non-canonical T3SS locus in other metapopulation strains. It is predicted to be complete and functional but unlike canonical T3SS loci, it lacks an identifiable *nod-*box upstream of a key regulator, suggesting it is not coregulated with *nod* genes. Vertical lines link pairs of *sctU* genes present within strains, as some have an additional non-symbiosis T3SS locus. The clade with homologs of the bacterial flagella is collapsed. Both trees are midpoint rooted. Branches colored in black exceed 95% UFBoot and 80% SH-aLRT support. Scale bars indicate average number of substitutions per site. Tip points of trees are colored according to the taxonomic classification of plant hosts and shaped according to the genomic location of the locus. Download FIG S2, PDF file, 0.1 MB. Copyright © 2022 Weisberg et al. 2022 Weisberg et al. https://creativecommons.org/licenses/by/4.0/ This content is distributed under the terms of the Creative Commons Attribution 4.0 International license.

10.1128/mbio.00074-22.3 FIG S3 Symbiosis MGEs are variable in gene composition. (A) Heatmap of gene presence/absence. Each column represents a cluster of homologous genes. A colored box indicates presence of the gene in the corresponding MGE (rows) while a white line indicates an absence. The dendrogram was generated based on the complete linkage method for clustering of binary distances. (B) Cophylo plot linking gene presence/absence patterns to strain phylogeny. The phylogeny is derived from the same data used to generate the trees in Fig. 3. Scale bars indicate average number of substitutions per site. Linking lines are colored according to the taxonomic classification of plant hosts. Dendrogram tip points are shaped according to the class or type of symbiosis MGE and colored according to the taxonomic classification of plant hosts. Download FIG S3, PDF file, 0.4 MB. Copyright © 2022 Weisberg et al. 2022 Weisberg et al. https://creativecommons.org/licenses/by/4.0/ This content is distributed under the terms of the Creative Commons Attribution 4.0 International license.

**FIG 2:**
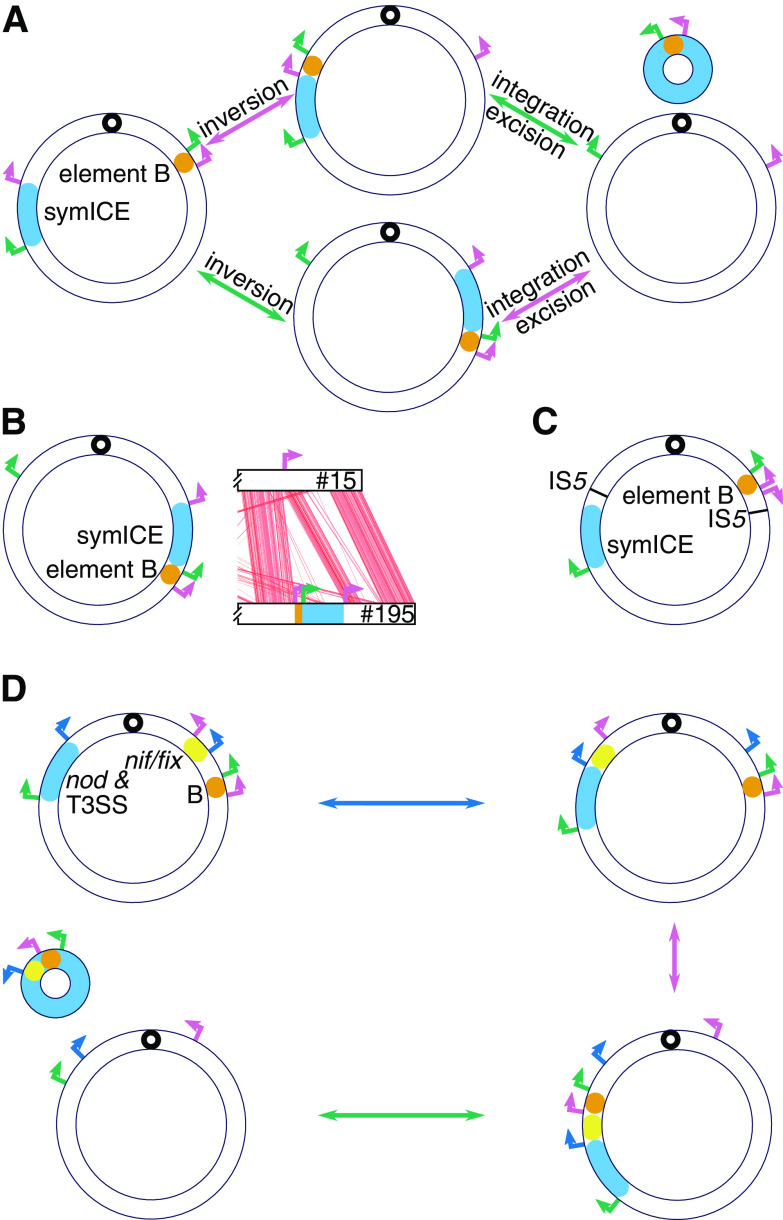
Recombination and organization of symICEs in chromosomes of *Bradyrhizobium* strains. (A) Model for recombination of the bipartite tRNA-Val symICE. Two recombination events are predicted to occur between *att* sites and result in the excision (left to right) or integration (right to left) of a symICE into a chromosome. Some recombination events are predicted to be associated with inversions to the genome. The order in which pair of *att* sites recombine determines whether recombination between the symICE and chromosome proceeds along the top or bottom path. (B) Organization of the bipartite tRNA-Val symICE in chromosome of strain number 195 (left is a model; right is an alignment of a portion of the chromosome to that of non-nodulating strain number 15; red lines link homologous regions). (C) Organization of the bipartite tRNA-Val symICE in the chromosome of strain USDA6. (D) One model for recombination (excision follows the clockwise path while integration follows the counterclockwise path) of the tripartite tRNA-Val symICE variant in strain number 186. Its *nif/fix* genes are split off to a region near a tRNA-His and are linked to an *intH* gene. In all panels, small black circles represent origins of replication. Single headed arrows are colored according to sequence and indicate locations and orientations of *att* sites. Double headed arrows indicate excision or integration, and are colored like the color of the reocmbining *att* sites.

Previous work used genomic comparisons, *att* site identification, and experimental transfer to characterize tripartite symICEs in *Mesorhizobium*, wherein sequential steps assemble the parts into a single circular element during recombination (13). Molecular evidence herein is consistent with symICEs of *Bradyrhizobium* having the capacity to mobilize and for some to induce large-scale rearrangements during recombination with chromosomes. The tRNA-Val (subtype A) symICE is the most common subtype. However, unlike those in *Mesorhizobium*, most of these adopt a bipartite configuration with a symICE element integrated near a tRNA-Val gene and a “B” element near the *ybgC* gene (Fig. 2). Each of the two elements has an integrase gene bordering a predicted *att* site and the other *att* site at the other boundary of the element; and recombination is predicted to occur in two steps, causing a chromosomal inversion around cognate *att* sites in each element (Fig. 2A; Fig. S1 and S4; Data set S1). This proposed mechanism of recombination is supported by alterations seen in four variants of tRNA-Val (subtype A). The tRNA-Val (subtype A) symICE of strain #195 is organized in a manner consistent with being in an intermediate configuration, as its two elements are adjacent to each other at one of the predicted *att* sites. Those of strain USDA6 and closely related E109 are predicted to be locked in the chromosome due to them having an *att* site transposed proximal to its partner *att* site, precluding its integrases from mediating recombination (Fig. 2B and C). Last is the symICE of strain number 186, which adopts a tripartite configuration (Fig. 2D). This tRNA-Val (subtype A) symICE acquired a third integrase and *att* site that resulted in *nif/fix* genes splitting into a third region adjacent to a tRNA-His gene. Similar to polypartite *Mesorhizobium* symICEs, this variant is predicted to require three steps to integrate and excise and cause two genome inversions (13). Four other types of symICEs are monopartite and likely integrate in one step at different tRNA genes.

10.1128/mbio.00074-22.4 FIG S4 The tRNA-Val symICEs are associated with genome inversions. Representative genomes of beneficial strains carrying tRNA-Val symICEs were aligned to representative genomes of non-nodulating strains (top and bottom) lacking any symICE. Positions (boxes) of the two elements of the bipartite tRNA-Val symICE are indicated. Orientation and location of *att* sites (arrows) are shown for the genomes of the non-nodulating strains. Red lines associate homologous regions. Download FIG S4, PDF file, 0.10 MB. Copyright © 2022 Weisberg et al. 2022 Weisberg et al. https://creativecommons.org/licenses/by/4.0/ This content is distributed under the terms of the Creative Commons Attribution 4.0 International license.

**FIG 3:**
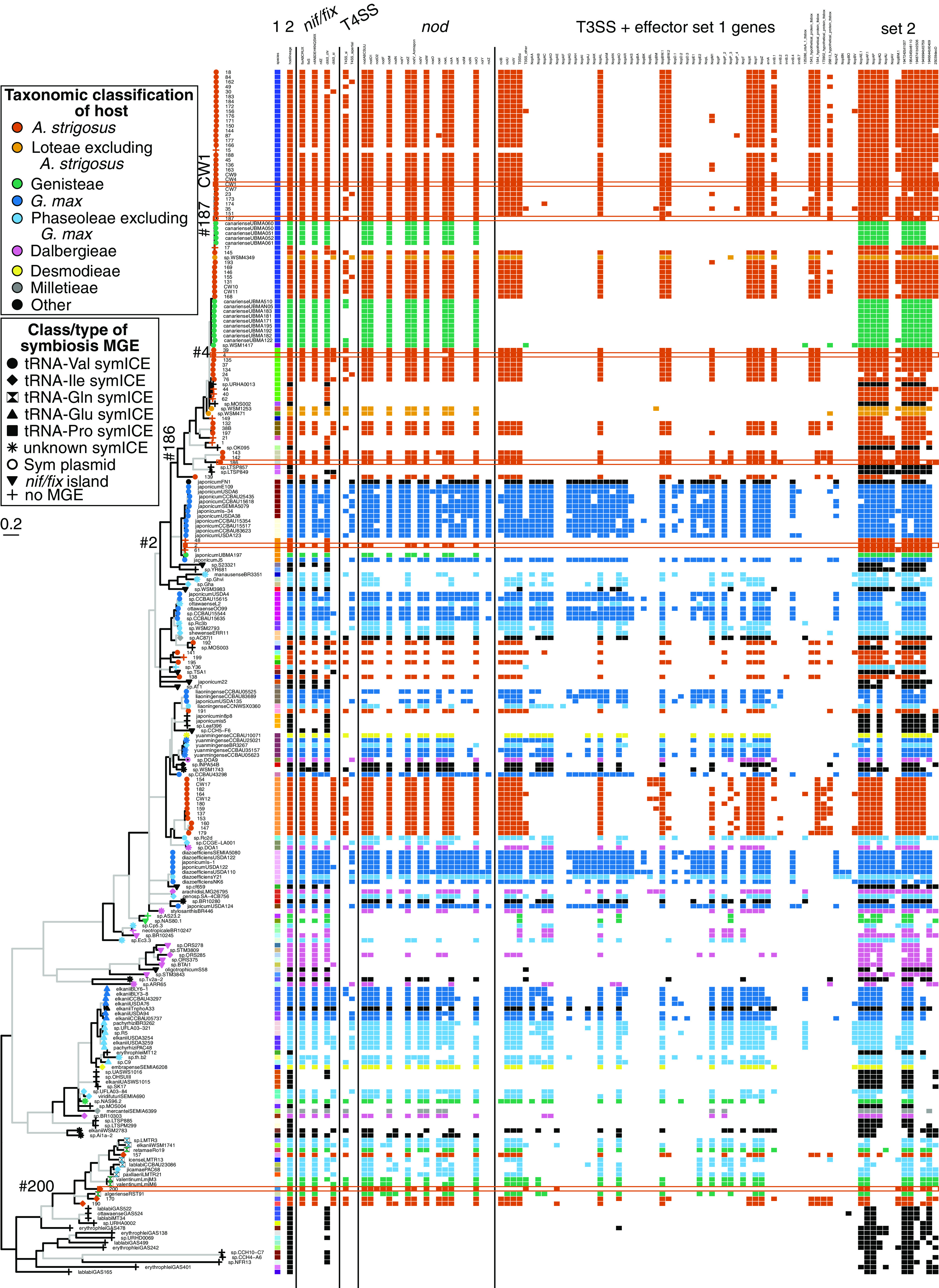
Diversity in combinations of *Bradyrhizobium* species and symbiosis MGEs. A multilocus sequence analysis (MLSA) maximum likelihood (ML) tree of *Bradyrhizobium* strains is shown on the left. The tree is midpoint rooted. Black colored branches exceed 70% bootstrap support. Gray colored branches have 51% to 70% bootstrap support. Branches with ≤ 50% bootstrap support were collapsed. The scale bar indicates average number of substitutions per site. Tip points are colored according to the taxonomic classification of plant hosts and shaped according to the class or type of symbiosis MGE. The next two columns show the species-level classification for strains (Extended Data set S2) and the associated class or type of symbiosis element. Remaining columns represent genes or functional gene clusters. A colored box indicates the presence in the corresponding symbiosis element. A white box indicates an absence. T3SS-associated effector genes are separated into two sets. Set 2 consists of predicted effector genes located in the chromosome, distal to symICEs.

Phylogenetic patterns of symbiosis MGEs are consistent with them having been mobilized horizontally across the genus (9, 10). Diverse species-level groups of *Bradyrhizobium* have similar symbiosis MGEs and are associated with the same host taxon while clades of closely related *Bradyrhizobium* vary in symbiosis MGEs and include ineffective and non-nodulating strains (Fig. 3; Fig. S3; Extended Data sets S1–S3). We examined conserved single copy genes in symbiosis MGEs to identify evidence for transmission events. Genes formed 14 groups based on similar evolutionary histories, reflecting substantial recombination and reshuffling (Fig. S5; Extended Fig. S2; Extended Data set S4). Nonetheless, symICE types and subtypes tended to cluster similarly across the 14 trees, each of which has a topology that differs from that of the genus tree, suggesting that symbiosis MGEs are largely inherited vertically and horizontally, as single units.

10.1128/mbio.00074-22.5 FIG S5 ML trees for 14 sets of concatenated genes defined on having similar tree topologies. The trees were ranked and numbered based on the greatest number of genes to the fewest number of genes. Key functions, genes, and total number of genes are listed below each tree. Trees are midpoint rooted. Branches colored in black exceed 95% UFBoot and 80% SH-aLRT support. Scale bars indicate average number of substitutions per site. Tip points of the trees are colored according to the taxonomic classification of plant hosts and shaped according to the genomic location of the genes. Download FIG S5, PDF file, 0.3 MB. Copyright © 2022 Weisberg et al. 2022 Weisberg et al. https://creativecommons.org/licenses/by/4.0/ This content is distributed under the terms of the Creative Commons Attribution 4.0 International license.

10.1128/mbio.00074-22.9 DATA SET S2 Universal, core, and signature genes. Download Data Set S2, XLSX file, 0.02 MB. Copyright © 2022 Weisberg et al. 2022 Weisberg et al. https://creativecommons.org/licenses/by/4.0/ This content is distributed under the terms of the Creative Commons Attribution 4.0 International license.

### Modularity of symbiosis MGEs enables diversification while preserving SNF.

**FIG 4:**
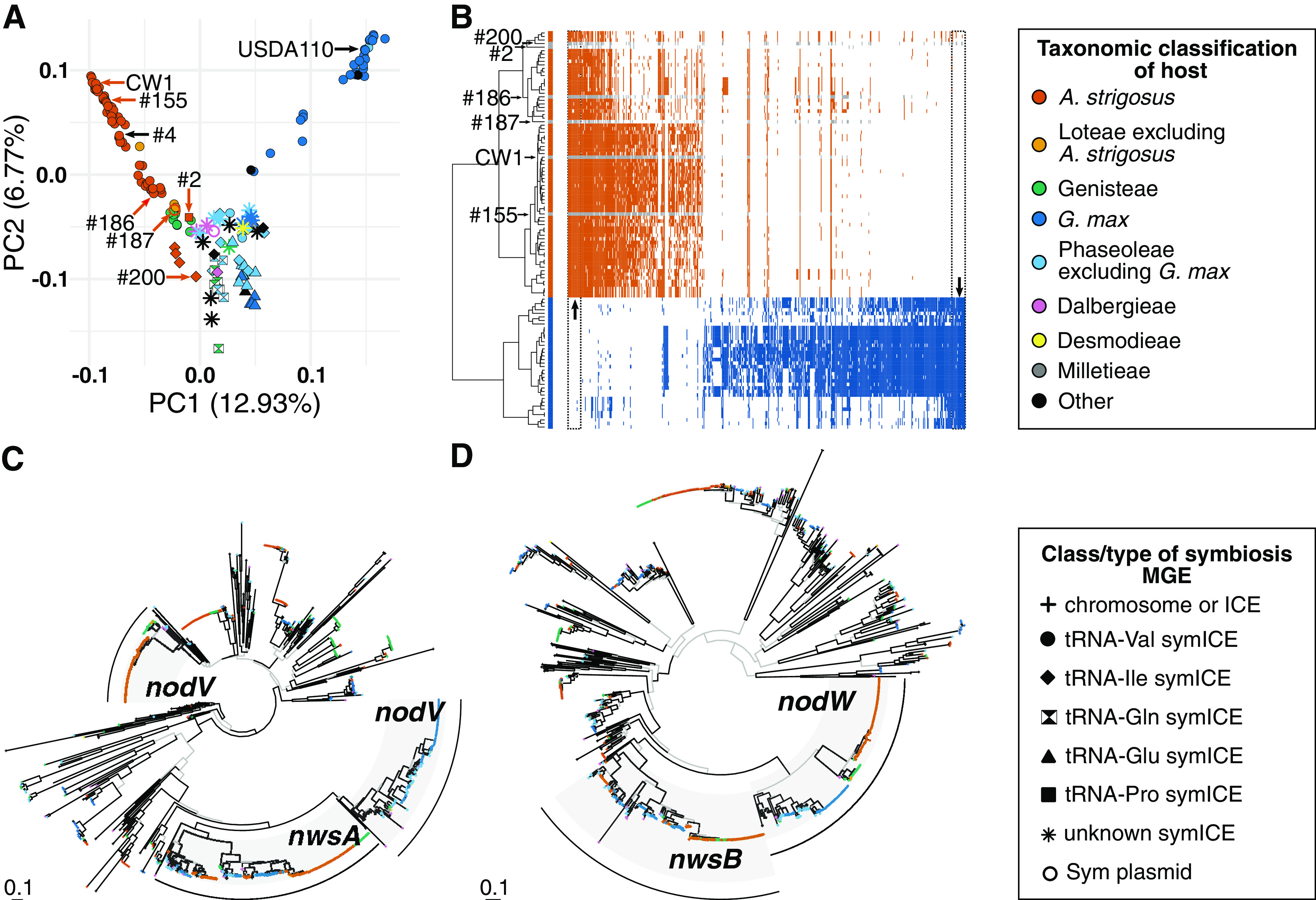
Symbiosis MGEs differ substantially in gene composition. (A) Principal-component analysis (PCA) of gene presence/absence of symbiosis MGEs. Each point represents a symbiosis MGE. Metapopulation strains that are ineffective on *A. strigosus* are labeled. Strains number 4 and USDA110 are shown as references. (B) A genome-wide association study was used to identify genes (columns) that are enriched in symbiosis MGEs (rows) of strains associated with *A. strigosus* or *G. max* (Data set S3). Shown are significant genes (Bonferroni corrected) and sorted based on enrichment in strains associated with *A. strigosus*. The two boxes outline regions with signature genes which were highly ranked, in ≥ 95% of the strains of the group, and in ≤ 5% of the comparator group. Gray colored rows correspond to ineffective strains. Ward’s distance of gene presence/absence patterns was used to hierarchically cluster symbiosis MGEs. Ineffective metapopulation strains are indicated. (C) and (D) Deep maximum likelihood phylogenetic gene trees for homologs of *nodV* and *nodW*, respectively. Clades with *nodV*, *nwsA*, *nodW*, and *nwsB* are labeled. Branches colored in black exceed 95% UFBoot and 80% SH-aLRT support. Scale bars indicate average number of substitutions per site. Data and tip points are colored according to the taxonomic classification of plant hosts and shaped according to the location of the gene or class/type of symbiosis MGE.

Like other rhizobia, most genes implicated in SNF of *Bradyrhizobium* are encoded within MGEs (9, 10, 13, 14, 16). Thus, transfer of MGEs and recombination of symbiosis genes could be important to inform on mechanisms that diversify and impact SNF functions. We used principal-component analysis (PCA) to visualize the compositional variation of the symbiosis MGEs and showed that a large proportion is associated with differences in host plant species (Fig. 4A). Two additional observations of metapopulation strains provided critical insights. First, symbiosis MGEs of ineffective strains are each more similar in composition to that of a beneficial strain than to each other, suggesting that symbiosis MGEs of ineffective strains are derived from those of beneficial strains. Second, both tRNA-Val (subtype A) and tRNA-Ile (subtype A) symICEs can confer benefit to *A. strigosus*, suggesting that symbiosis genes have been shuffled across symICE types. Collectively, these observations indicate that modularity impacts both loss and gain of plant species as hosts.

10.1128/mbio.00074-22.10 DATA SET S3 GWAS results. Download Data Set S3, XLSX file, 0.1 MB. Copyright © 2022 Weisberg et al. 2022 Weisberg et al. https://creativecommons.org/licenses/by/4.0/ This content is distributed under the terms of the Creative Commons Attribution 4.0 International license.

To identify reshuffling events that are associated with ineffectiveness, we first grouped genes of the symbiosis MGEs into three categories, those representing the minimal set necessary for symbiotic nitrogen fixation, e.g., *nif/fix*, and *nodABC*, those core to strains beneficial to *A. strigosus*, and those identified in a genome-wide association study defined as signatures of being associated with *A. strigosus* relative to Glycine max (Data set S2). The genes within these categories were then used to query for differences in ineffective strains. Based on comparisons of genes essential for SNF present in beneficial *Bradyrhizobium*, the symbiosis MGEs of the six ineffective strains number 2, number 155, number 186, number 187, number 200, and CW1 are predicted to have genes necessary and sufficient for SNF but relative to those of beneficial metapopulation strains, have gene polymorphisms predicted to affect specificity of *A. strigosus* as a host species (Fig. 3; Fig. 4B; Fig. S2B; Fig. S6; Data sets S2–S3) (22). For example, the symbiosis MGEs of strains number 2, number 187, and number 200 lack symbiosis-associated T3SS and effector genes and several signature genes associated with being a symbiont of *A. strigosus.* Other differences in sequences or composition of *nod* genes and other genes in symbiosis MGEs were also identified (Fig. S6A; Fig. S7A–C, Data sets S2 and S3; Extended Data set S5) (27–29).

10.1128/mbio.00074-22.6 FIG S6 Symbiosis MGEs of metapopulation strains are related. (A) Synteny plot of symbiosis MGEs from representative strains (underlined strains are ineffective on *A. strigosus*). Key functional gene clusters are represented by different colored boxes. Strains can have up to four clusters (1, 2, 3-1, and 3-2) of *nif/fix* genes that correspond to a single cluster in symbiosis MGEs (1), a set of *nif/fix* genes in a *nod* cluster (2), and *fixNOQP* and *fixGHIS* located distal (3-1) or within (3-2) to symbiosis MGEs. They are distinguished by empty boxes or presence of leftward or rightward directed hash marks. The tRNA-Ile symICEs have two clusters of distantly related *nod* genes (empty green box versus green box with hash marks) and strain number 200 has a distantly related T3SS loci located distal to its symICE (orange box with hash marks). The B element is not shown for the tRNA-Val symICEs. Black boxes represent other functional gene clusters present in symICEs. Double hash marks indicate a separation of regions in the chromosome. The plasmid in strain number 187 is depicted as a linear molecule. Blue bars represent symbiosis MGEs of metapopulation strains. Gray bars depict representative symbiosis MGEs of nonmetapopulation strains. (B) Gene synteny graph of linear symICE elements of tRNA-Val symICEs from six metapopulation strains. The T4SS is within the variable region of symICEs and proximal to a recombination hotspot. Despite being flanked by highly variable regions, T4SS genes clustered into only two topology groups inferred to have shared evolutionary histories and the topologies of their trees are consistent with the grouping of symICE types. (C) Gene synteny graph showing conservation of Sym regions and divergence of variable regions in linear symICE elements of tRNA-Val from strains number 184 (species group 13) and number 141 (species group 30). In (B and C), paths consist of nodes which represent clusters of homologous genes (key functional gene clusters are colored and/or labeled). Yellow colored nodes correspond to insertion sequences and transposase-encoding genes. Colored arrows are used to indicate the two *att* sites. Download FIG S6, PDF file, 0.3 MB. Copyright © 2022 Weisberg et al. 2022 Weisberg et al. https://creativecommons.org/licenses/by/4.0/ This content is distributed under the terms of the Creative Commons Attribution 4.0 International license.

10.1128/mbio.00074-22.7 FIG S7 Variation in *nod* genes associated with host specificity (A). A cophylo plot linking strain phylogeny to *nodA* phylogeny. Arrows are used to indicate strains with multiple *nod* genes. The second copy of *nodA* in strain DOA9 is diverged and did not sufficiently align to be included in the tree. The trees rotated to minimize differences in tree structures. In the MLSA tree, branches with ≤ 50% bootstrap support were collapsed. Tip points of the MLSA tree are colored according to the taxonomic classification of plant hosts. Tip points of the *nodA* tree are colored similarly and shaped according to the class or type of symbiosis MGE. (B) ML tree for *nodD1.* The pseudogenized allele in strain number 200 and second set of homologs present in tRNA-Ile (subtype A) symICEs, are indicated. Inspection of Illumina and Nanopore reads for strain number 200 independently corroborated a frameshift mutation at position 167 and a predicted downstream premature stop codon. (C) ML tree for *nodF.* The homolog in strain number 200 is indicated. All trees are midpoint rooted. Black colored branches have 70% or more bootstrap support. Gray colored branches have less than 70% bootstrap support. Scale bars indicate average number of substitutions per site. Tip points of the trees are colored according to the taxonomic classification of plant hosts and shaped according to class or type of symbiosis MGE. Download FIG S7, PDF file, 0.1 MB. Copyright © 2022 Weisberg et al. 2022 Weisberg et al. https://creativecommons.org/licenses/by/4.0/ This content is distributed under the terms of the Creative Commons Attribution 4.0 International license.

**FIG 5:**
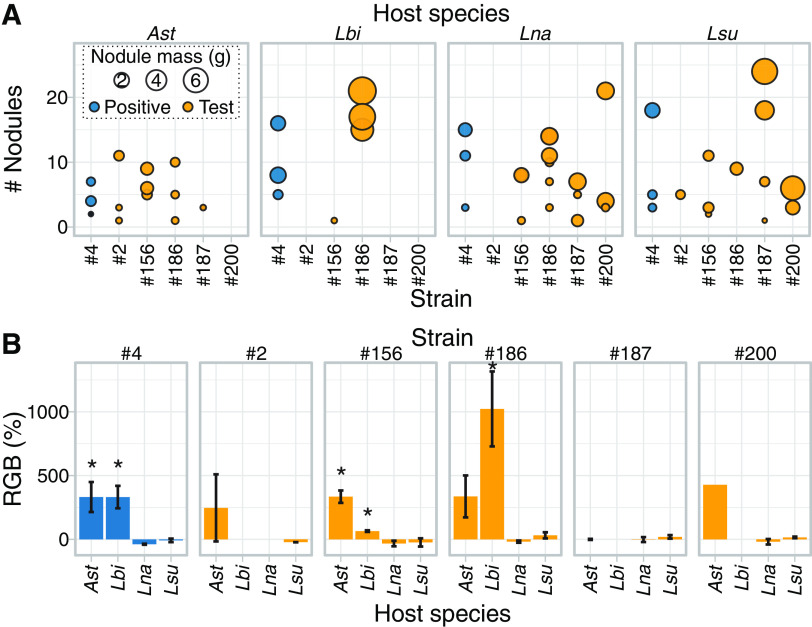
Strains ineffective on *A. strigosus* can nodulate and benefit other plant species. (A) Balloon plots relating number and total dry mass of nodules in four plant species infected with strains of *Bradyrhizobium*. Only plants with a minimum of two nodules were included in analyses. *A. wrangelianus* was tested as a partner species but did not form nodules and is not shown. (B) Relative growth benefit (RGB) provided by six strains of *Bradyrhizobium* on four species of plants. Relative growth is expressed as percent change in biomass compared to the biomass of plants from the corresponding host species treated with only water. A “*” indicates a significant relative change (*P*-value ≤ 0.05). Abbreviations used are: *Ast* = *A. strigosus*, *Lbi* = *L. bicolor*, *Lsu* = *L. succulentus*, and *Lna* = *L. nanus*. Measurements are provided (Extended Data set S6).

To test predictions that ineffective strains maintain SNF functions but benefit different plant species, we measured symbiosis outcomes on five sympatric legume species (Extended Data set S6). Four ineffective strains, marginally beneficial strain number 156, and beneficial strain number 4 can all nodulate multiple host species that they overlap in their native range (Fig. 5A). On *A. strigosus*, benefits derived from strain number 186 were not significant, consistent with its classification as ineffective (22). However, strain number 186 exhibited a qualitative shift in host range by inducing nodules on *Lupinus bicolor.* This strain caused significant growth benefits that were strikingly greater than those caused by strains number 4 and number 156, which also caused significant beneficial effects to *L. bicolor* (Fig. 5B). These data suggest that shuffling of symbiosis genes preserve SNF functions while changing host specificity, thus providing an explanation for the maintenance of variation in effectiveness in metapopulation strains.

We also uncovered processes in the evolution of symbiosis MGEs predicted to have allowed metapopulation strains to gain *A. strigosus* as a host species and provide benefits. First, *nodV* was identified as a signature gene in the GWAS analysis. NodVW is a two-component system that induces *nod* gene expression upon sensing specific plant isoflavones (30). In all sequenced strains, the *nodV* and *nodW* genes are adjacent to each other in the symICE. In addition, *nodVW* are close paralogs of *nwsAB* in strain USDA110, a symbiont of *G. max* and other hosts in the Phaseoleae tribe (30). The phylogeny of *nodV* is polyphyletic and incongruent with the phylogeny of *nodW*, supporting a scenario where a distantly related *nodV* homolog was acquired by an ancestral tRNA-Val symICE (Fig. 4C and D). We predict that it displaced the original and maintained the ability to function with the original *nodW*, thereby gaining a new capacity to perceive *A. strigosus* as a host species while retaining the ability to regulate *nod* gene expression.

Second, we predict that reshuffling of genes among symICEs led to strains gaining *A. strigosus* as a host species. Our analysis suggested a tRNA-Ile symICE of a symbiont associated with a host outside the Loteae tribe (i.e., *Acmispon*, *Lotus*, related genera) acquired a region from a tRNA-Val (subtype A) symICE, yielding the tRNA-Ile (subtype A) symICE with two *nod* clusters (Fig. S2B. Fig. S6A, S7). The acquired region includes most of the *nod* genes common to beneficial metapopulation strains, including *nodVW* with the *A. strigosus-*associated *nodV* allele. In beneficial strains, the region includes symbiosis-associated T3SS- and effector-encoding genes but in ineffective strain number 200, they are absent. The nonfunctional *nodD1* allele of strain number 200 also resides in this region. Across tRNA-Ile (subtype A) symICEs, the acquired region lacks a complete set of *nod* genes necessary for host-specific nodulation and normal nodule development (30). However, it is complemented by the other *nod* region, and three of four strains with a tRNA-Ile (subtype A) symICE can benefit *A. strigosus*. Thus, modularity allows genes to reconfigure at different levels of organization and diversify *Bradyrhizobium* strains without compromising essential functions of SNF.

### Reorganization of symICE modules promotes diversification.

**FIG 6:**
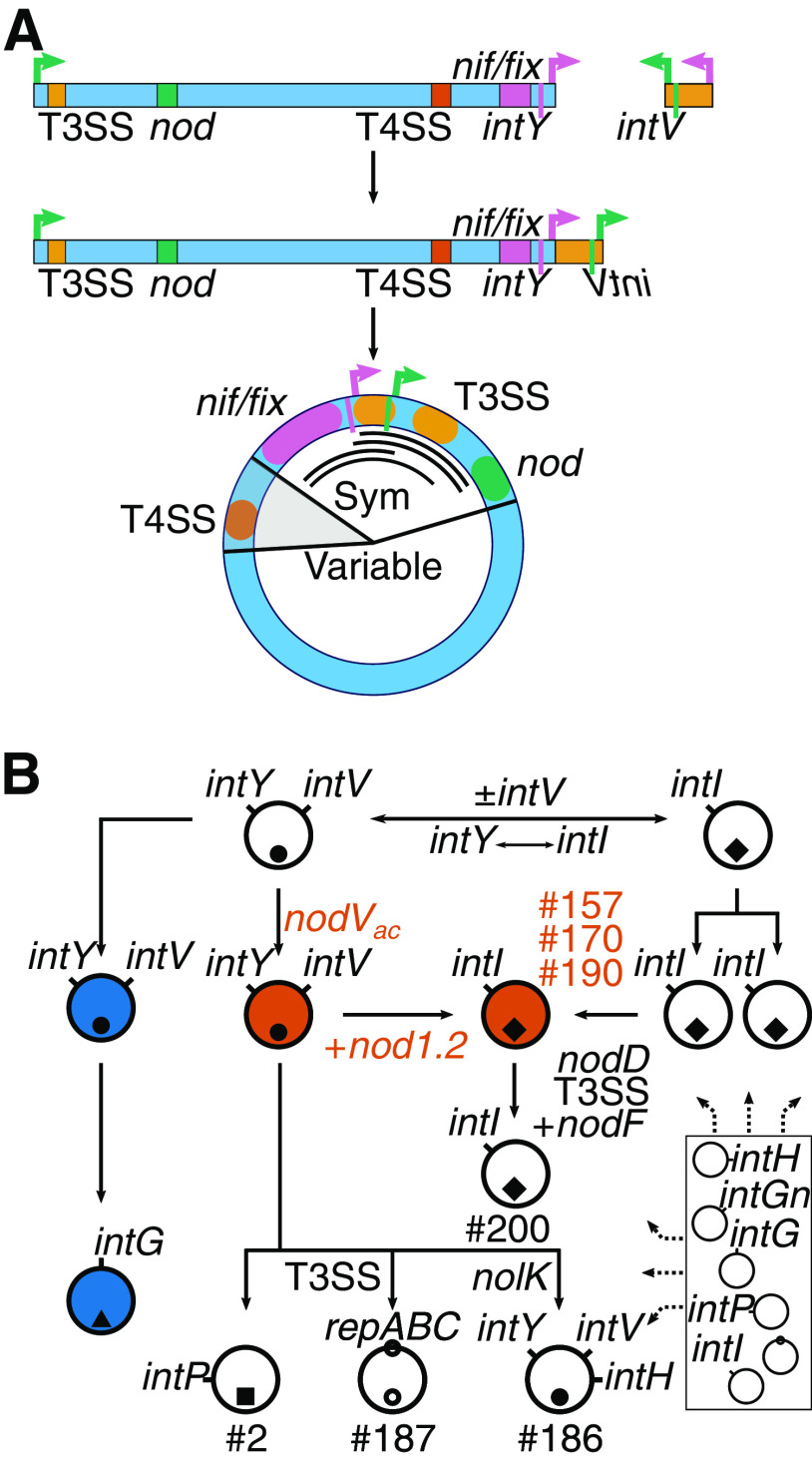
Model for the evolution of *Bradyrhizobium* symbiosis MGEs. (A) Circularization of tRNA-Val symICEs occurs in two steps and reorganizes genes. Black arcs in the middle of the circularized symICE delineate (from outside to inside) regions absent from the Sym plasmid of strain number 187, tRNA-Pro symICE of strain number 2, tRNA-Ile symICE (subtype A) of three beneficial strains, and the tRNA-Ile symICE (subtype A) of strains number 200. Key functional gene clusters, *att* sites, integrase genes, and the variable region are indicated; the left half is more ambiguous, as indicated by the gray colored area (see Fig. S6B and C). (B) Relationships among symbiosis MGEs and other MGEs in *Bradyrhizobium*. Large circles represent symbiosis MGEs (vermillion = *A. strigosus-*associated; blue = *G. max-*associated) and small symbols within them represent their type. Integrase genes are abbreviated with “*int*” and a letter denoting the location of its attachment site. The boxed region in the lower right represents the accessory genome with collections of symbiosis and nonsymbiosis MGEs.

The process by which symICEs excise causes important changes to gene organization that inform on mechanisms that led to the diversity of SNF uncovered herein (Fig. 6). Upon circularization, *Bradyrhizobium* symICEs reorganize SNF-associated genes into a small “Sym region,” including *nif/fix*, T3SS-encoding, and *nod* genes (13) (Fig. 6A; Fig. S6B–C). We predict that the reconfiguration, with SNF-associated genes more closely clustered and separated from a large variable region, is crucial for reshuffling across elements and generating the diversity observed in symbiosis MGEs among metapopulation strains. We suggest that the Sym region recombines with other elements, as the *Bradyrhizobium* genus is predicted to have an extremely large and diverse collection of nonsymbiosis associated MGEs (Fig. 1 and 4; Extended Fig. S3). For example, the Sym region from a tRNA-Val (subtype A) symICE likely recombined with a tRNA-Pro ICE and plasmid to yield the symbiosis MGEs in strains number 2 and number 187, respectively (Fig. 6B; Fig. S1, S2A, and S6A). Likewise, evidence strongly supports a scenario where the Sym region recombined with a tRNA-Ile symICE to yield the A subtype (Fig. 6B; Fig. S6A). Recombination of Sym regions also occurred among tRNA-Val symICEs and ICEs, generating members with highly similar symbiosis genes and diverged variable regions (Fig. S5 and S6B–C). Consistent with this, the variable regions of examined tRNA-Val symICEs have closely related T4SS-encoding loci flanked by large polymorphic regions, nested within closely related Sym regions (Fig. S2A and S6B–C). However, evidence also suggests that in other symbiosis MGEs, recombination can occur across more distantly related molecules and lead to the acquisition of more divergent T4SS-encoding loci and potentially, its flanking variable regions (Fig. S2A).

The model of symICE excision predicts that joining and circularization of bipartite tRNA-Val symICEs will link integrase genes together with *att* sites and the small B element. This subsequent predicted reorganization is critical in facilitating losses of this region, whose absence is necessary to transition one type of symICE to another that integrates at a different site, or to a Sym plasmid that does not integrate into the chromosome. The Sym plasmid of strain number 187 has a scar, one *att* sequence of a tRNA-Val symICE, supporting the proposed loss. Furthermore, loss of one or both integrase genes is consistent with the emergence of tRNA-Ile symICEs from a tRNA-Val symICE (Fig. 6B; Fig. S1B, and S1F). Critically, losses can also include genes flanking the B element and impact SNF. Loss of T3SS-encoding genes and signature genes is associated with three strains exhibiting ineffectiveness on *A. strigosus* (Fig. 6; Fig. S6A). Conversely, loss of most of the *nif/fix* genes from the donor tRNA-Val symICE appeared to have occurred in the transition of tRNA-Ile (subtype A) symICE to benefit *A. strigosus* while loss of both flanking regions resulted in ineffective strain number 200. The flanking region affected depends on whether recombination is with another symICE or with a nonsymbiosis ICE, as loss of *nif/fix* is not selected against if recombination is with an element that already confers SNF.

However, organizational differences can also restrict recombination between symICEs. Subtypes A and B of the tRNA-Val symICEs, associated with symbiosis on Loteae and Phaseoleae hosts, respectively, are derived from a common ancestor, but there is little evidence for them recombining (Fig. 4A; Fig. S1A–B and S5). When tRNA-Val (subtype B) symICEs circularize, *nod* and T3SS-encoding genes will separate to opposite regions flanking the B region, as opposed to the same region predicted for subtype A. We suggest this difference reduces productive recombination events between these two subtypes (Fig. S6A).

## DISCUSSION

Here, we showed that modularity and reshuffling of genes by mobile genetic elements generate uncooperative genotypes of rhizobia and make individual partnerships unstable, but these same properties are fundamental for robustness and extending beneficial associations to diverse host species, as well as transferring symbiotic capacity among diverse rhizobia. We sampled native bacteria from an 800 km transect of wild *Acmispon strigosus* populations and included strains various in symbiosis phenotypes. We applied a strategy, developed for virulence plasmids, to study symbiosis ICEs and plasmids in *Bradyrhizobium*, and in doing so we were able to infer relationships among many symbiosis MGEs and model interactions among MGEs that led to their diversification (31). Findings suggested that symICE circularization reorganizes genes and is predicted to promote the shuffling of blocks of SNF genes into different ICE backbones, the generation of new combinations of modules, the acquisition of genes from the chromosome onto the symICE, and transfer of the SNF trait to plasmids. In this regard, despite being integrated in chromosomes for most of their life cycle, symICEs are like plasmids in promoting more rapid evolution of their cargo genes (32). The symICEs described here are incredibly diverse, and even metapopulation strains isolated from a single plant host species and sampled in one US state can have different symICE types, subtypes, or symbiosis plasmids. No two symICEs isolated from metapopulation strains or in this data set have identical gene content or sequence. This reflects multiple scales of modularity. Plants can be host to diverse species of *Bradyrhizobium*, strains can host diverse and exchangeable symbiosis MGEs, which themselves exchange and acquire genes, and individual genes within functional units can vary.

The patterns that we uncovered in the metapopulation strains, including the role of MGEs in gene reshuffling and diversification, extend broadly across the *Bradyrhizobium* genus. For example, most analyzed photosynthetic nitrogen fixing *Bradyrhizobium* strains have only *nif/fix* islands and are restricted in host range (25, 33). But two strains acquired symICEs that expanded their host ranges. Notably, strain ORS285 has an island that we suggest is a remnant of a symICE located at tRNA-Ile that includes *nod*, T3SS-associated genes, and an integrase gene but no *nif/fix* genes or conjugation machinery-encoding locus (26) (Fig. S1B and S2B). Moreover, several variants of symICEs have also recurrently gained *fixNOQP* and *fixGHIS*, genes necessary for respiration in microoxic root nodules and typically in *fix* gene cluster III located in the chromosome (34) (Extended Fig. S4). Acquisition of these symICE variants extended SNF to strains that lack *fix* gene cluster III and would not otherwise be capable of supporting SNF. Thus, acquisition of MGEs, and the traits that they encode, appear to play a major role in the adaptation of *Bradyrhizobium* to novel lifestyles.

We propose that diversification in the *Mesorhizobium* genus, which exhibits many parallel patterns, is also driven by acquisition and reshuffling of MGEs (19). Importantly, key aspects of our study differ from those of *Mesorhizobium*, where diversification has been primarily characterized in managed systems. In *Mesorhizobium*, entire symICEs originating from inoculum strains were predicted to be transferred into indigenous nonsymbiotic rhizobia, or among strains already naturalized under monoculture crops, a scenario that imposes intense selection for host crop compatibility (14, 16, 17, 19). Conversely, our findings are based on investigation of phenotypically variable strains of *Bradyrhizobium* isolated from diverse native plant communities, where multiple legume species overlap and select for differential subsets of rhizobia (35–37). Our study suggested that symICE transfer has recurrently promoted novel host acquisition, and that loss of effectiveness on one host is associated with gains of other hosts, processes that likely require a diverse array of potential hosts.

Reconceiving symbiosis as a dynamical system with links that can form and dissolve among symbionts and between symbionts and hosts is essential for revealing emergent properties. Modularity and flexibility of genetic elements, coupled to their mobility, drive diversification, giving rise to variation in symbiosis, such as that revealed in *Bradyrhizobium.* Modularity and flexibility are central to robustness (3). A fundamental principle of robustness is that it maintains the function of a system, not a state (38). Therein lies the source of the paradox where symbiosis functions are maintained at the system level but lost from individual states, such as a symbiont, partnership, or host (2). Models that reduce symbiosis to bipartite partnerships and ignore symbiont-symbiont interactions unknowingly neglect major sources of variation and overlook robustness (2, 39). Additionally, by separating symbiosis into categorical partnerships, these models fail to recognize the effects of multiple and various symbiont-host interactions within the system. Host species select for different combinations of symbiosis genes in their bacterial partners. Pangenome evolution, shaped by individual and gene-level selection, reassorts genes into new combinations that can extend symbiosis to new host species. Hence, alignment of fitness interests between host and symbiont is necessary for persistence of a partnership while interactions diverse in partners are necessary for robustness and evolutionary stability of symbiosis (3).

This alternative framework provides a predictive understanding of symbiosis functions that are encoded on MGEs. SNF is evolutionarily stable despite repeated abandonment by both symbiont and host species (22, 40). Conversely, symbioses involving vertically transmitted endosymbiotic bacteria with closed pangenomes are not as robust and are at higher risks of extinction (41). In agriculture, elite rhizobia strains are often added to monocultures in attempt to establish a highly specific and optimal partnership. Success is difficult to achieve because the system is flexible, and plants can partner with different genotypes of rhizobia (9). Even if the optimal partnership is attained, the likelihood for it to persist is low because of potential trade-offs between state optimality and system robustness (42). Strategies that promote interactions between multiple lineages of beneficial nitrogen-fixing rhizobia and diverse crops will have greater success for long-term sustainability.

## MATERIALS AND METHODS

### Genome sequencing, assembly, and annotation.

Strains were selected from a metapopulation that was previously generated and phenotyped (22, 23) (Data set S1). Bacteria were grown overnight in a modified arabinose-gluconate medium at 29°C with shaking (43). The Qiagen DNeasy blood and tissue kit (Qiagen, Valencia, CA, USA) was used to extract total genomic DNA, and according to methods previously described, prepared, multiplexed, and sequenced on one channel of an Illumina HiSeq 3000 (Illumina Inc., San Diego, CA USA) by the Center for Genome Research and Biocomputing (CGRB; Oregon State University, Corvallis, OR, USA) to generate 150mer paired end sequencing reads (31).

For long read sequencing, strains were either sequenced on two flow cells of an Oxford Nanopore GridION X5 by De Novo Genomics Corporation (Kansas City, KS, USA) or on a MinION flowcell (1D chemistry, LSK109) on a Mk1b MinION sequencer controlled by a MinIT coprocessor. In both cases, samples were multiplexed and prepared using a pooled 1D Native genomic DNA library prep (SQK-LSK109) prior to sequencing (44). Previously described methods were used to process and assemble sequencing reads and annotate genome assemblies (31).

BLASTN v. 2.6.0 searches were used, with “TTCACACGGGAGAGGTCCAAGGTTCGATCCCTTGTGCGCCCACCATTCACCT” and “TCCGTATCTTCGAAATAGACGCGGACCTGCATATGATGGTGACCGCCGCGAATTTCGCCATCAAGAGAAGCTGTCACG” as queries and the parameters “–word_size 7 –evalue 100” to identify attachment sequences of Int_V_ and Int_Y_, respectively (45). HMMER v 3.3 hmmsearch and custom hmm profiles were used to annotate putative *nod*-box and *ttsI-*box sequences (46–48). Macsyfinder 1.0.5 with the default options and the hmm profiles TXSS and CONJ were used to annotate secretion system-encoding loci (49). BLASTP and translated sequences of type III effector genes from rhizobia were used as queries to identify homologs of effector genes (48, 50).

### Analyses of genome sequences.

Previously described methods were used to calculate percentage of conserved proteins (POCP; 50% threshold) and pairwise average nucleotide identity (ANI; threshold ≥ 95%) to operationally classify strains into genus- and species-level groups, respectively (31). The software package get_homologues v. 20170418 with the options “-M -t 0” was used to cluster genes into orthologous groups (51). Gene presence/absence heatmaps were generated using the R package heatmap.plus with “complete” clustering of binary distances, or with the ggtree function gheatmap (52, 53). Publicly available genome sequences were downloaded from NCBI on May 7, 2018.

BWA-MEM, Picard tools, and GATK HaplotypeCaller, following previously described methods, were used to call single nucleotide polymorphisms (SNPs) (31, 54, 55).

Panaroo v. 1.2.3 with the option “—clean-mode sensitive” was used to generate a pangenome graph for select genomes, which was visualized using Cytoscape v. 3.8.0 (56, 57).

Islander v. 1.2 with the options “–translate –trna –annotate –reisland –table 11 –nocheck” was used to identify tRNA-associated ICEs in finished or hybrid assembled genome sequences (58). Islander v. 1.2 was modified by increasing maximum island size thresholds to 2 Mb to search for large symICEs. Predicted regions, conserved and syntenic across multiple strains and spanning large clusters of tRNA loci, were identified manually and filtered out.

Plasmids were identified from finished or hybrid assembled genome sequences as separate replicons or from draft genome sequences as contigs with a *repABC* locus.

### Analysis of symICE and symbiosis genes.

The symICEs in finished or hybrid assembled genome sequences were identified based on presence of *nod*, T3SS-associated, and *nif*/*fix* genes. The program progressiveMauve was used to identify symICE boundaries by comparing genome sequences with those from closely related strains that lack the same type of symICE types (59). The symICEs were classified into types based on concordance with identified ICE regions, location of tRNA genes, and the presence of integrase genes next to or overlapping border sequences. Repetitive sequences at border regions, including putative *att* sites were also identified. Sequences corresponding to symICEs of draft genome sequences were identified by using CONTIGuator v. 2.7, with default parameters, to map contigs to complete genome sequences with most similar *nif*/*fix* genes (60). Contigs and regions mapping within the symICE region of the reference genome were extracted.

A symICE gene presence/absence matrix, representing the diversity of gene content of symICEs, was inferred from the ortholog clusters of the full genome get_homologues analysis. The ortholog group for each symICE-associated gene was identified from the larger analysis and using the “spread” function of the R package tidyr, a new presence/absence matrix containing only symICE genes and paralogs was generated (61). These new gene cluster sequences were subset in fasta format and used in phylogenetic and topology clustering analysis. The base R function “prcomp” was used to perform a principal-component analysis (PCA) of symICE gene content (62). Scoary v. 1.6.16 with the options “-s 4 –collapse -e 100” was used to perform a genome wide association study (GWAS) analysis of symICE gene content, comparing symICEs of strains isolated from *A. strigosus* and *G. max* (63). LAST v. 1066 lastal with the option “-f BlastTab+” was used to compare symICEs, and homologous regions were visualized using the BioPython package GenomeDiagram v. 1.72 (64, 65). A previously described method was used to generate and visualize gene synteny networks for select symICEs (31).

Sourmash v. 2.0.0a11 with the option “compute –scaled 100” and “compare -k 21” was used to estimate a Jaccard Index between symbiosis MGEs based on their *k*-mer signatures (66). These values were used to build graphs where symbiosis MGEs are nodes, and edges connect them in which at least one symbiosis MGE has a Jaccard Index ≥ 0.1 to the other. Cytoscape v. 3.8.0 was used to visualize graphs (56).

### Construction of phylogenetic trees.

The translated sequences of *dnaG*, *frr*, *infC*, *nusA*, *pgk*, *pyrG*, *rplA*, *rplB*, *rplC*, *rplE*, *rplF*, *rplK*, *rplL*, *rplM*, *rplN*, *rplP*, *rplS*, *rplT*, *rpmA*, *rpoB*, *rpsB*, *rpsC*, *rpsE*, *rpsI*, *rpsJ*, *rpsK*, *rpsM*, *rpsS*, *smpB*, and *tsf* were used to construct a multilocus sequence analysis (MLSA) phylogeny (67, 68). Previously described methods were followed to construct phylogenetic trees and for phylogenetic topology clustering analysis (31). IQ-TREE v. 1.6.12 with the options “-bb 1000 -alrt 1000” was used to generate phylogenies for some data sets (69). Cophylo plots were generated using the R package phytools (70).

The top 100 hits of a BLASTP search, using SctU as a query against the NCBI nr database, was retrieved on March 4, 2019. BLASTP with the default parameters was used to identify homologs of *sctU*, *nodV*, *nodW*, *repC*, and *trbE* in the genomes of analyzed strains. Data sets for integrases and other gene/protein phylogenies were comprised of ortholog groups from the get_homologues analysis.

### Plant inoculations.

*Bradyrhizobium* strains number 2, number 4, number 156, number 186, number 187, and number 200 were each inoculated on to five sympatric host species, including *A. strigosus* AcS049, *A. wrangelianus* AcW10-R5, and *L. bicolor*, *L. succulentus*, *L. nanus*. Inbred lines were used for *Acmispon* whereas mixed seed sets of *Lupinus* were used (S & S Seeds, Carpinteria, CA). Seeds were surface sterilized in 5.0% sodium hypochlorite, rinsed in sterile water, and scarified. For *A. wrangelianus*, seeds were vernalized for a week at 4°C before planting (71). For others, immediately after scarification, seeds were planted in sterilized containers (Steuwe and Sons, Corvallis, OR), each filled with autoclaved Turface Pro League soil mixed 1:1 with small- and coarse-grain sand. The seedlings were germinated in a growth facility and when true leaves appeared, moved to a greenhouse. Plants were hardened to greenhouse conditions for 1 week before inoculation.

Previously published protocols were followed to inoculate plants with *Bradyrhizobium* strains (43). A minimum of six plant replicates was used for each host-strain combination. Negative-control plants received sterilized reverse-osmosis H_2_O only. Immediately before inoculation, plants were arranged in a randomized design blocking by plant size, determined based on the number of true leaves. Plants were fertilized weekly with 5.0 mL nitrogen-free Jenson’s fertilizer. After 7 weeks postinoculation, plants were depotted, rinsed of remaining soil, and photographed. We counted and weighed nodules and measured separately dry biomasses of shoots and roots. Tissues were separated and dried at 60°C for at least 72 h prior to weighing.

The effect of strain inoculation on plants was calculated as percent relative growth benefit (RGB), the mean percent biomass of an inoculated plant relative to its corresponding control plants:

RGB = 100 * (Inoculated host biomass-control host biomass)/control host biomass.

Linear models were used to investigate variation in nodulation (total nodule number, dry nodule biomass) and RGB for effects of strain, host, and interaction effects. We used the same model for all tests to improve normality of the residuals and homoscedasticity. Significant differences among strains or hosts were assessed using Tukey’s HSD test. To test if RGB was significantly larger than zero, we performed a series of one sample unpaired t-tests. All statistical analyses were carried out using R version 3.6.0 (62).

### Data and materials availability.

Short reads and assemblies have been deposited in NCBI as BioProject PRJNA671608 and accession numbers are listed in Data set S1. Network graphs in nexus or sif format, phylogenetic trees in Newick format, genome annotations, and scripts can be downloaded from https://github.com/osuchanglab/BradyrhizobiumPangenomeManuscript. Strains sequenced in this study are available from JLS upon request. Extended supplementary figures and data sets can be downloaded from https://github.com/osuchanglab/BradyrhizobiumPangenomeManuscript/tree/main/Extended_Supplementary_Materials.
