# Supplementary material for: Pangenome Evolution Reconciles Robustness and Instability of Rhizobial Symbiosis
Source: mBio. 2022 Apr 13;13(3):e00074-22. doi: 10.1128/mbio.00074-22 (PMC9239051; doi:10.1128/mbio.00074-22)
Supplement: FIG S1 [file mbio.00074-22-s0001.pdf]

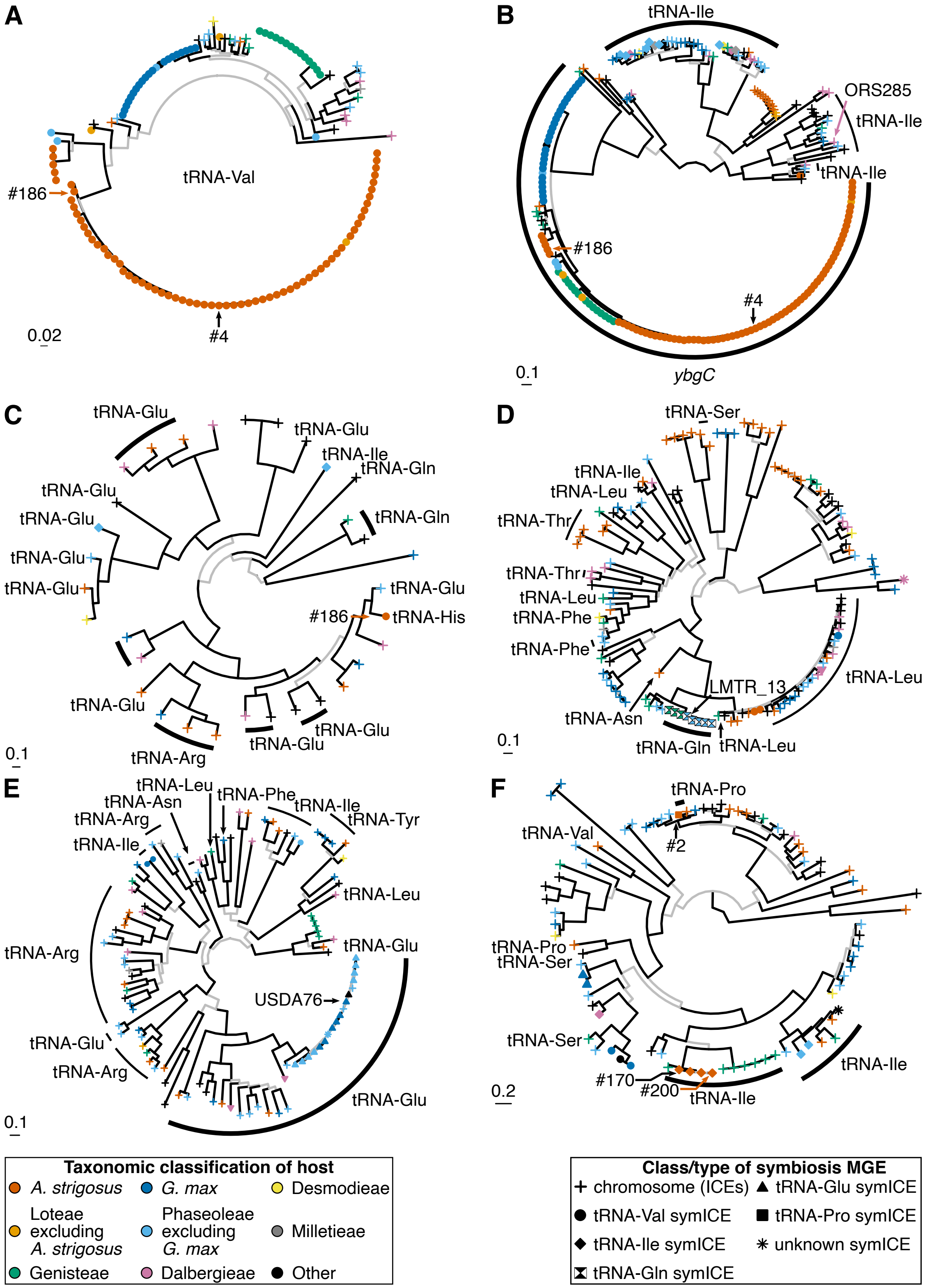
A
tRNA-Val
#186
#4
0.02
B
tRNA-Ile
ORS285
tRNA-Ile
tRNA-Ile
#186
#4
0.1
ybgC
C
tRNA-Glu
tRNA-Glu
tRNA-Ile
tRNA-Gln
tRNA-Glu
tRNA-Glu
tRNA-Gln
tRNA-Glu
tRNA-Glu
tRNA-Glu
#186
tRNA-His
tRNA-Glu
tRNA-Glu
tRNA-Glu
tRNA-Arg
0.1
D
tRNA-Ser
tRNA-Ile
tRNA-Leu
tRNA-Thr
tRNA-Thr
tRNA-Leu
tRNA-Phe
tRNA-Phe
LMTR_13
tRNA-Leu
tRNA-Asn
tRNA-Gln
tRNA-Leu
0.1
E
tRNA-Leu
tRNA-Asn
tRNA-Phe
tRNA-Ile
tRNA-Arg
tRNA-Tyr
tRNA-Ile
tRNA-Leu
tRNA-Arg
tRNA-Glu
USDA76
tRNA-Glu
tRNA-Arg
tRNA-Glu
0.1
F
tRNA-Pro
tRNA-Val
#2
tRNA-Pro
tRNA-Ser
tRNA-Ser
#170
#200
tRNA-Ile
tRNA-Ile
0.2
Taxonomic classification of host
A. strigosus
G. max
Desmodieae
Loteae excluding A. strigosus
Phaseoleae excluding G. max
Milletieae
Genisteae
Dalbergieae
Other
Class/type of symbiosis MGE
chromosome (ICEs)
tRNA-Glu symICE
tRNA-Val symICE
tRNA-Pro symICE
tRNA-Ile symICE
unknown symICE
tRNA-Gln symICE
