# Supplementary figures and images for: Pangenome Evolution Reconciles Robustness and Instability of Rhizobial Symbiosis
Source: mBio. 2022 Apr 13;13(3):e00074-22. doi: 10.1128/mbio.00074-22 (PMC9239051; doi:10.1128/mbio.00074-22)

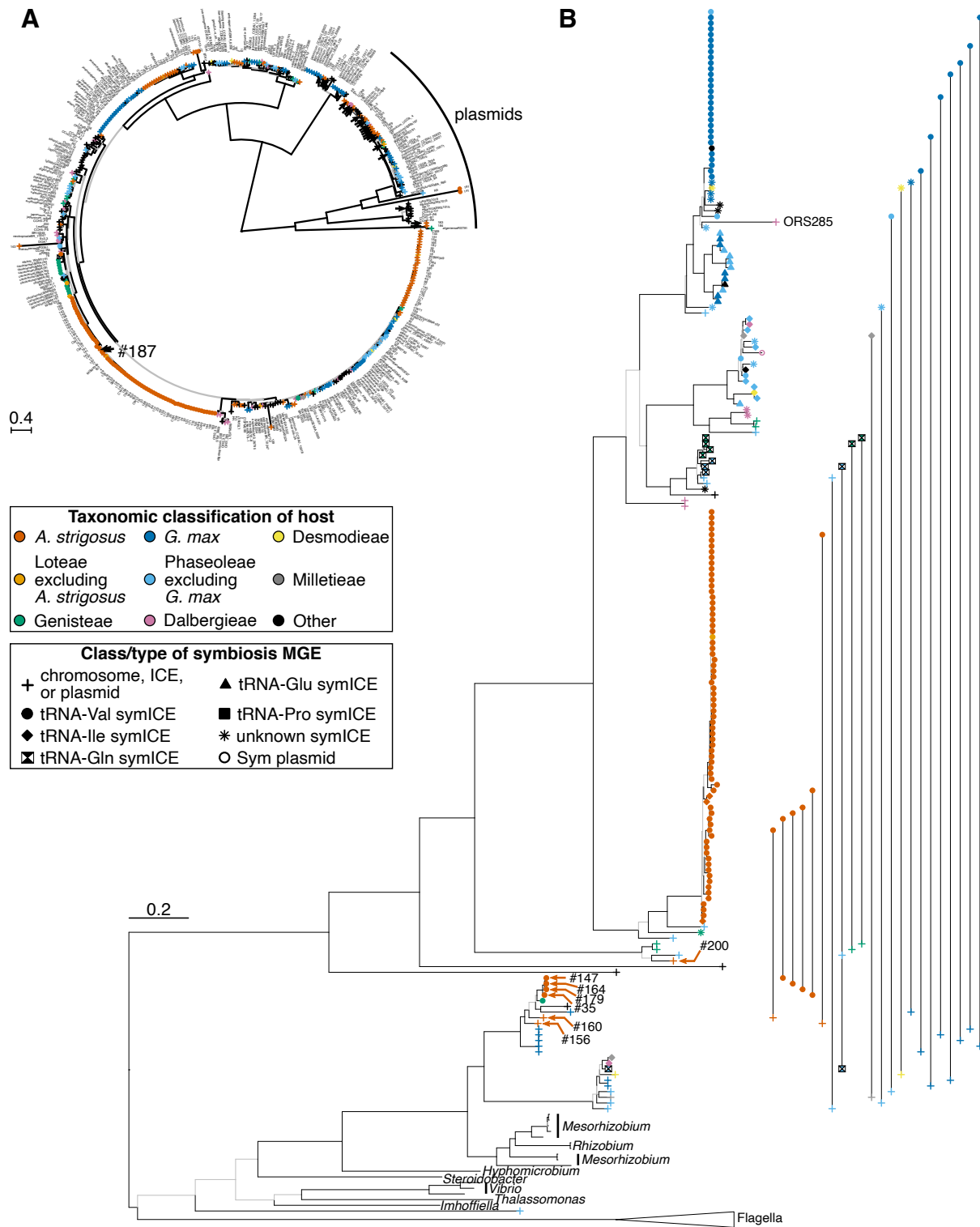

Supplement: FIG S2 [file mbio.00074-22-s0002.pdf]

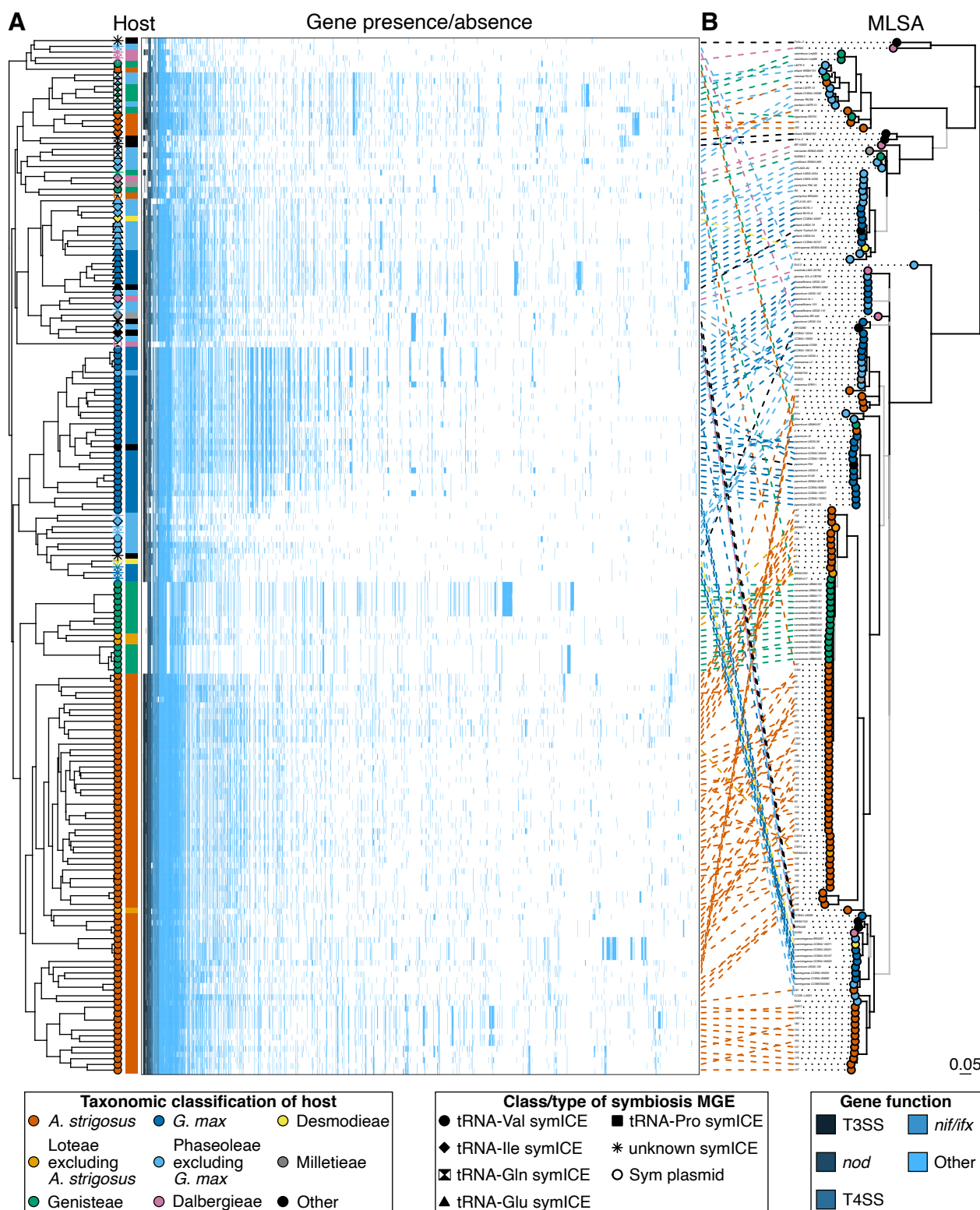

Supplement: FIG S3 [file mbio.00074-22-s0003.pdf]

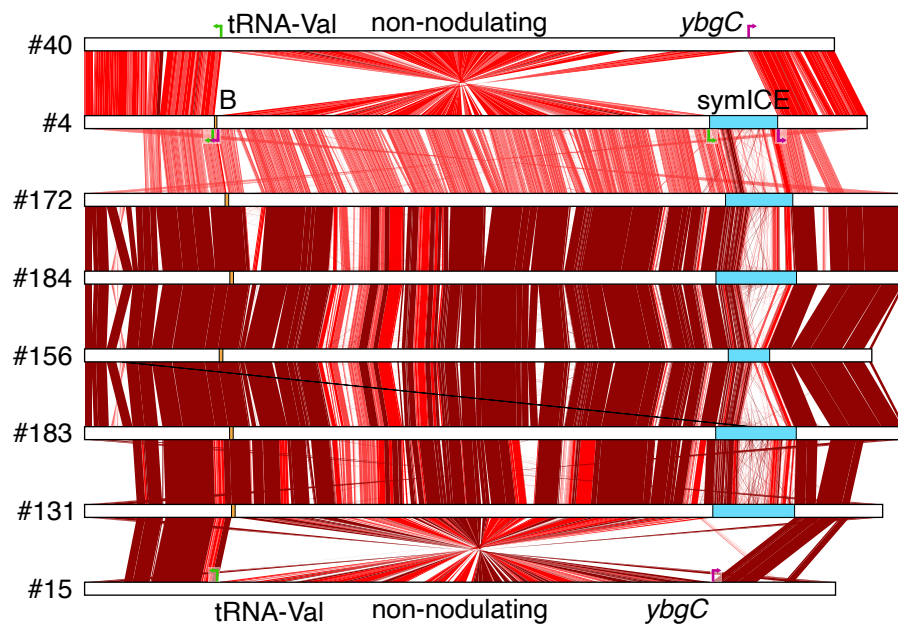

Supplement: FIG S4 [file mbio.00074-22-s0004.pdf]

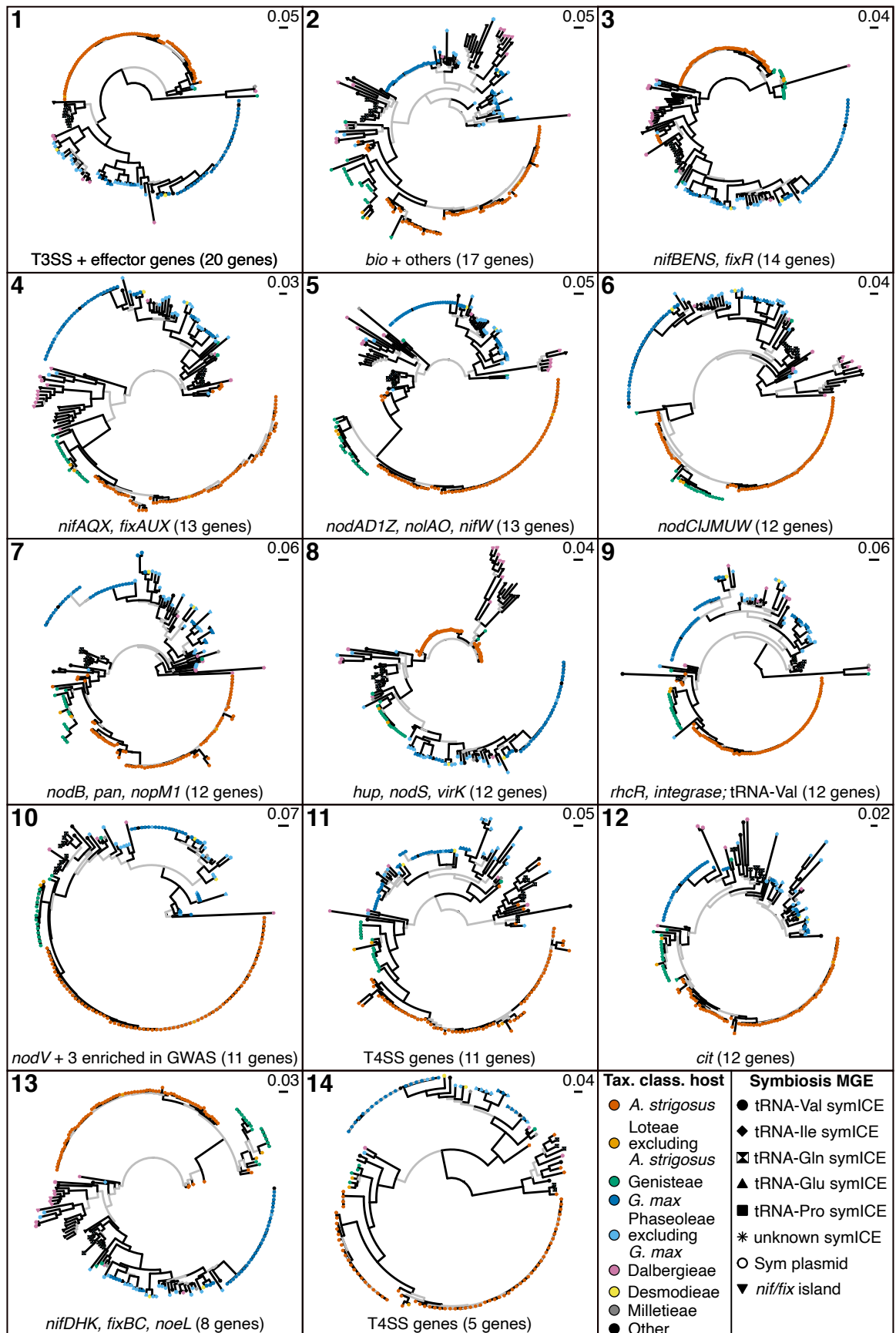

Supplement: FIG S5 [file mbio.00074-22-s0005.pdf]

**A**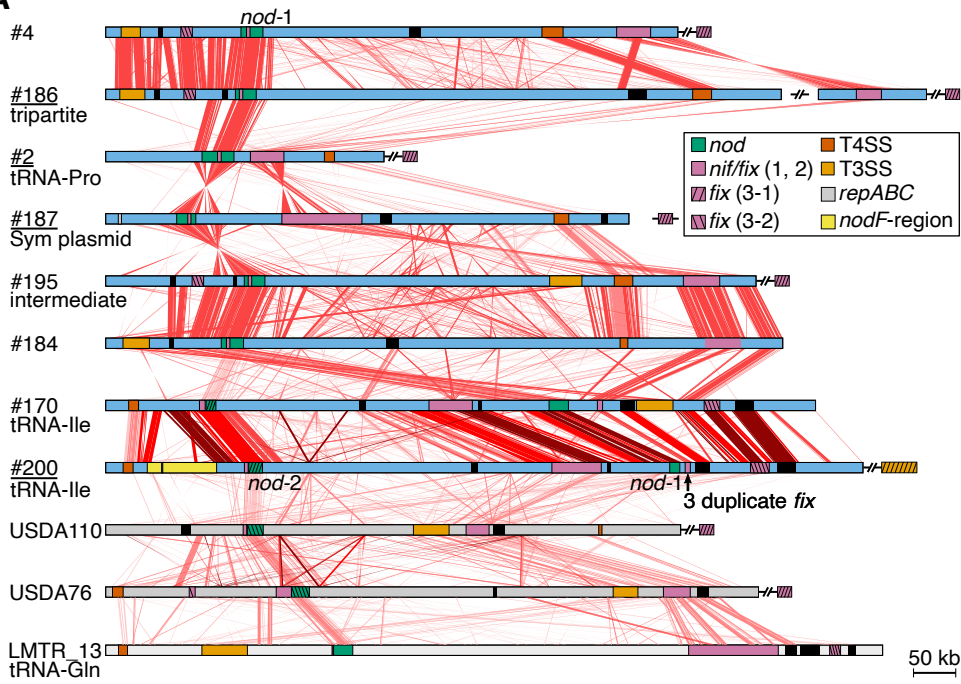**B**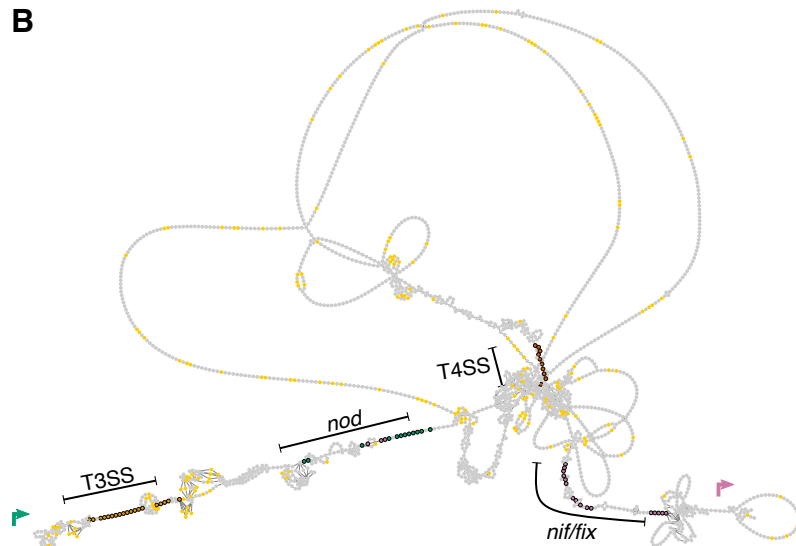**C**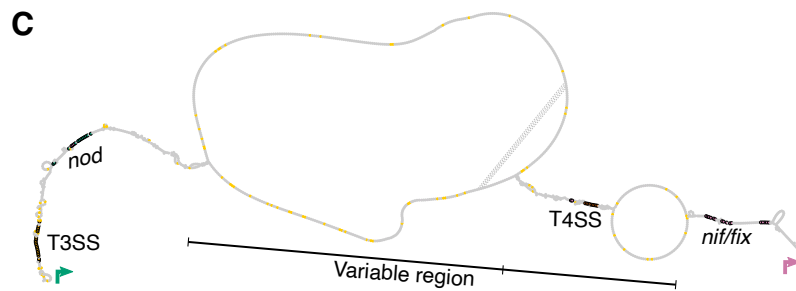

Supplement: FIG S6 [file mbio.00074-22-s0006.pdf]

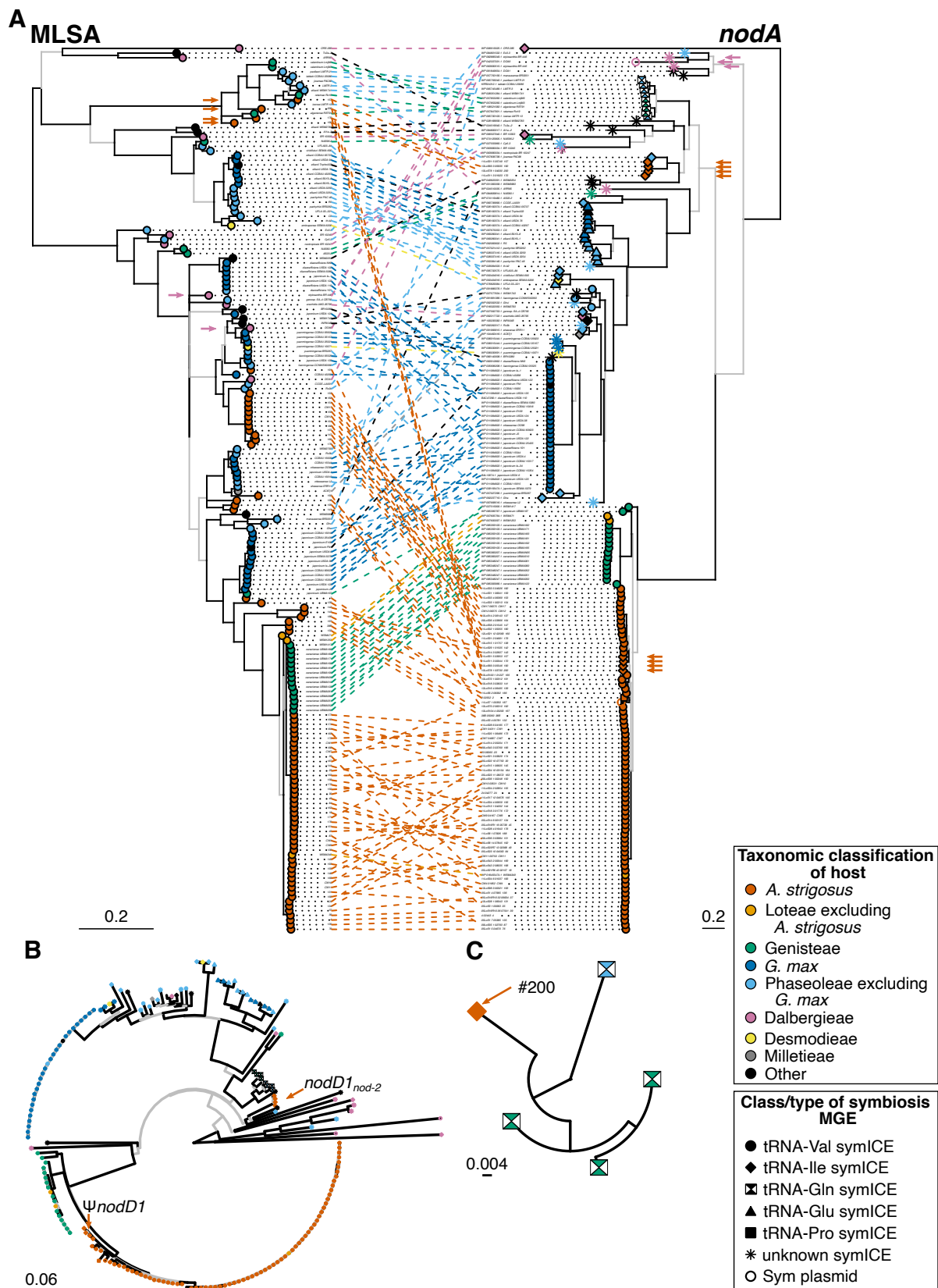

Supplement: FIG S7 [file mbio.00074-22-s0007.pdf]
